# Autosomal Recessive Primary Microcephaly: Not Just a Small Brain

**DOI:** 10.3389/fcell.2021.784700

**Published:** 2022-01-17

**Authors:** Sami Zaqout, Angela M. Kaindl

**Affiliations:** ^1^ Department of Basic Medical Sciences, College of Medicine, QU Health, Qatar University, Doha, Qatar; ^2^ Biomedical and Pharmaceutical Research Unit, QU Health, Qatar University, Doha, Qatar; ^3^ Institute of Cell and Neurobiology, Charité—Universitätsmedizin Berlin, Berlin, Germany; ^4^ Center for Chronically Sick Children (Sozialpädiatrisches Zentrum, SPZ), Charité—Universitätsmedizin Berlin, Berlin, Germany; ^5^ Department of Pediatric Neurology, Charité—Universitätsmedizin Berlin, Berlin, Germany

**Keywords:** MCPH genes, microcephaly, brain, intellectual disability, neuronal differentiation, animal models, brain malformation

## Abstract

Microcephaly or reduced head circumference results from a multitude of abnormal developmental processes affecting brain growth and/or leading to brain atrophy. Autosomal recessive primary microcephaly (MCPH) is the prototype of isolated primary (congenital) microcephaly, affecting predominantly the cerebral cortex. For MCPH, an accelerating number of mutated genes emerge annually, and they are involved in crucial steps of neurogenesis. In this review article, we provide a deeper look into the microcephalic MCPH brain. We explore cytoarchitecture focusing on the cerebral cortex and discuss diverse processes occurring at the level of neural progenitors, early generated and mature neurons, and glial cells. We aim to thereby give an overview of current knowledge in MCPH phenotype and normal brain growth.

## Introduction

Microcephaly is clinically defined by a significant reduction of the occipito-frontal head circumference (OFC) of more than two (microcephaly) or three (severe microcephaly) SDs below the mean for a given sex, age, and ethnicity (von der Hagen et al., 2014). The prevalence of microcephaly ranges between 1.5 and 8.7 per 10,000 births in Europe and the United States, respectively (Cragan et al., 2016; Morris et al., 2016). However, 15%–20% of children with developmental delay have microcephaly (Sassaman and Zartler, 1982; Watemberg et al., 2002; Aggarwal et al., 2013). Depending on the time of appearance, microcephaly can be classified as primary/congenital or secondary/postnatal (Passemard et al., 2013; Woods and Parker, 2013; Zaqout et al., 2017). It has been suggested that the primary causes of microcephaly lead to a reduction in the number of generated neurons, while the secondary causes mainly affect the dendritic complexity and synaptic formations (Woods, 2004). Primary microcephaly is by definition present at birth, and it can be caused by environmental and/or genetic factors (Zaqout et al., 2017; Alcantara and O'Driscoll, 2014; Kaindl et al., 2010). Various environmental factors such as infections, toxins, radiation, or alcohol result in primary microcephaly. The recent identification of epidemic infections with the Zika virus as a cause for primary microcephaly has highlighted this rare condition as a key topic in neuroscience to understand normal brain development (Kleber de Oliveira et al., 2016; Subramanian et al., 2019). This condition is an addition to the genetic prototype of isolated primary microcephaly, autosomal recessive primary microcephaly (microcephaly primary hereditary (MCPH)).

MCPH is a group of rare heterogeneous neurodevelopmental disorders characterized by intellectual disability and a significant reduction in the brain volume reflected by a reduction in the head circumference already at birth (Kaindl et al., 2010; Zaqout et al., 2017; Slezak et al., 2021). The reduction in brain volume in MCPH cases affects disproportionately the neocortex, though without obvious changes in the cortical organization (Kaindl et al., 2010; Kraemer et al., 2011; Jayaraman et al., 2018). The increasing use of whole-exome sequencing (WES) has uncovered a growing number of novel and disease-causing MCPH variants (Boycott et al., 2013). Simultaneously, further radiological and postmortem studies expand the spectrum of brain malformations reported in individuals with MCPH. The prevalence of MCPH differs from 1:10,000 in populations with a high rate of consanguineous marriage to 1:250,000 in the general population (Van Den Bosch, 1959; Cox et al., 2006). In consanguineous families, most MCPH diagnosed cases reveal homozygous variants in the disease-causing gene. However, compound heterozygous variants are increasingly discovered in MCPH patients, raising the importance of using advanced and accurate diagnosis methods for such cases (Jean et al., 2020).

Currently (December 2021), twenty-eight MCPH-related genes have been identified and tagged sequentially as MCPH1–MCPH28 (MCPH; OMIM phenotypic series: PS251200; Siskos et al., 2021) (Table 1). Still, more genetic loci are expected to exist given the fact that approximately 62% of western Europeans/North Americans and 25% of Indians/Pakistani families diagnosed with MCPH fail to show linkage to any of the MCPH loci (Verloes et al., 1993; Kaindl et al., 2010; Sajid Hussain et al., 2013). Most *MCPH* gene variants are nonsense, frameshift, or splice site-affecting variants leading to a production of non-functional, truncated proteins (Kaindl et al., 2010; Barbelanne and Tsang, 2014; Jean et al., 2020). Most of the MCPH genes encode centrosomal and/or pericentriolar matrix (PCM) proteins that are, in turn, ubiquitously expressed (Kaindl et al., 2010; Hussain et al., 2013; Barbelanne and Tsang, 2014). It is therefore not surprising to find that many MCPH proteins are involved in centriole biogenesis including organization, maturation, and distribution (Subramanian et al., 2019; Jean et al., 2020). Furthermore, MCPH proteins play crucial roles in microtubule dynamics, mitotic spindle formation, DNA damage responses, Wnt signaling, transcriptional regulation, and cell cycle checkpoint control (Kraemer et al., 2011; Mahmood et al., 2011; Jayaraman et al., 2018; Jean et al., 2020). Disruption of one or more of these functions during cortical neurogenesis adversely affects neuronal progenitor proliferation, differentiation, and survival leading to a severe reduction in the total number of generated neurons reflected by the microcephaly phenotype. Being highly conserved among species, ongoing research on MCPH animal models deems to be an important key for understanding the pathomechanisms behind microcephaly as well as the role of MCPH proteins during normal brain development (Gilbert et al., 2005; Woods et al., 2005; Zaqout et al., 2017). Although microcephaly found in MCPH patients simulates an evolutionary retrogression of the brain size (McHenry, 1994), human brain evolution cannot be attributed solely to the protein-coding sequences of MCPH genes (Pervaiz et al., 2021). Therefore, it has been hypothesized that complex conditional effects of human-specific coding and non-coding regulatory changes in MCPH only assist this evolution process (Pervaiz et al., 2021).

**TABLE 1:** List of microcephaly primary hereditary (MCPH) genes and related animal/organism models.

Locus	Protein	Gene	Location	OMIM	Model organisms	Generation method	Key findings	Ref
MCPH1	Microcephalin 1	*MCPH1*	8p23.1	607117	*Xenopus*	1. *Drosophila in vitro* expression cloning IVEC (DIVEC)	1. dMCPH1 is a substrate of anaphase-promoting complex (APC)	Hainline et al. (2014)
					Fly	2. Deletion of the *mcph1* gene by imprecise excision of a P-element	2. Lethal phenotype due to mitotic arrest, uncoordinated centrosome, and nuclear cycles	Brunk et al. (2007)
					Rodent	3. *Mcph1*-knockout mice (deletion of exon 4–5)	3. Premature increase in asymmetrical neural progenitor cell (NPC) divisions, uncoupled mitosis and centrosome cycle, misoriented mitotic spindle alignment	Gruber et al. (2011)
						4. *Mcph1*-knockout mice (gene trap)	4. Shorter survival rates, defected mitotic chromosome condensation	Trimborn et al. (2010)
						5. *Brit1*-knockout mice (gene targeting)	5. Hypersensitive to γ-irradiation, defective DNA repair, infertility, meiotic defects	Liang et al. (2010)
MCPH2	WD-repeat-containing protein 62	*WDR62*	19q13.12	613583	Fish	1. Morpholino-mediated knockdown of *wdr62*	1. Reduction in head and eye size, prometaphase delay, increased apoptosis	Novorol et al. (2013)
					Rodent	2. *Wdr62*-knockout mice (gene trap)	2. Abnormalities in asymmetric centrosome inheritance, neuronal migration delays, altered neuronal differentiation, prometaphase delay, infertility	Sgourdou et al. (2017)
						3. *Wdr62*-knockout mice (gene trap)	3. Mitotic arrest, cell death, reduced thickness of upper cortical neuronal layers, dwarfism	Chen et al. (2014)
						4. ShRNA knockdown of *Wdr62* in rats (*in utero* electroporation)	4. Premature differentiation of NPCs, abnormal spindle formation, and mitotic division	Xu et al. (2014)
						5. SiRNA knockdown of *Wdr62* in mice (*in utero* electroporation)	5. Spindle orientation defects, delayed mitotic progression, reduced NPC proliferation, increased cell cycle exit	Bogoyevitch et al. (2012)
						6. *Wdr62*-knockout mice (*Wdr62* ^*f/f*^; homologous recombination followed by germline transmission)	6. Mild microcephaly, reduced NPC number, impaired mitosis, increased apoptosis, increased cilium length	Zhang et al. (2019a)
					Human cerebral organoid	7. *WDR62* ^*−/−*^ cerebral organoids (mutant Human pluripotent stem cell (hPSC) lines; CRISPR-Cas9)	7. Reduced organoid size, reduced outer radial glial cell (oRGC) proliferation, impaired mitosis, increased NPC vertical division, premature differentiation, increased apoptosis, increased cilium length	
MCPH3	Cyclin-dependent kinase 5 regulatory subunit-associated protein 2	*CDK5RAP2*	9q33.2	608201	Fly	1. *Centrosomin* (*cnn*) knockout flies (chemical mutagenesis)	1. Nuclear cleavage defects, microtubule organization defects, abnormal mitotic spindle formation	Megraw et al. (1999)
							Disconnections between centrioles and PCM	Lucas and Raff, (2007)
					Rodent	2. *Hertwig’s anemia* mouse (inversion of exon 4 of *Cdk5rap2*)	2a. Fewer total neurons with special reduction in upper cortical neurons, abnormal spindle formation, and mitotic division, defective mitotic spindle orientation, premature cell cycle exit, increased cell death	Lizarraga et al. (2010)
							2b. Reduced dendritic complexity of layer 2/3 pyramidal neurons, increased spine density, shifted excitation—inhibition balance toward excitation	Zaqout et al. (2019)
						3. shRNA knockdown of *Cdk5rap2* in mouse (*in utero* electroporation)	3. Premature differentiation of NPCs, reduced proliferation, increased cell cycle exit	Buchman et al. (2010)
					Human cerebral organoid	4. RNAi knockdown of *CDK5RAP2* (co-electroporating green fluorescent protein (GFP) with shRNAs) and patient-derived cerebral organoids	4. Premature neural differentiation, increased NPC oblique, and vertical divisions	Lancaster et al. (2013)
MCPH4	Kinetochore scaffold 1	*KNL1*	15q15.1	609173	Rodent	1. Conditional *Knl1* knockout in mouse brain	1. Impaired NPC proliferation, missegregated chromosomes, DNA damage and p53 activation, rapid and robust apoptosis	Shi et al. (2019)
MCPH5	Abnormal spindle-like, microcephaly associated protein	*ASPM*	1q31.3	605481	Fish	2. Morpholino-mediated knockdown of *aspm*	2a. Reduction in head and eye size, prometaphase delay, increased apoptosis	Novorol et al. (2013)
							2b. Reduction in head and eye size, mitotic arrest, increased apoptosis	Kim et al. (2011a)
					Fly	3. Mutagenesis (x-irradiation)	3. High mitotic index, metaphase arrest, mitotic and meiotic non-disjunction, hemi-spindles formation	Gonzalez et al. (1990)
						4. Mutagenesis (recombinant chromosomes)	4a. Arrested mitotic cycle at metaphase, high frequency of polyploid cells, defected sex chromosome disjunction	Ripoll et al. (1985)
							4b. Disrupted microtubule-organizing centers, failure of cytokinesis	Riparbelli et al. (2002)
					Rodent	5. esiRNA knockdown of *Aspm* in Tis21–GFP knockin mice (*in utero* electroporation)	5. Centrosome detachment, altered cleavage plane orientation, increased non-NE fate, increased neuron-like fate	Fish et al. (2006)
						6. *Aspm*-knockout mice (gene trap)	6. Mild microcephaly, midbody localization defects, Major germline defects	Pulvers et al. (2010)
						7. *Aspm*-knockout mice (removal of exons 2 and 3)	7. Much thicker layer I and thinner layer VI cortical neurons, aberrant expression of Tbr1 and Satb2 in the subplate	Fujimori et al. (2014)
					Ferret	8. *Aspm* germline knockout ferret	8. Severe microcephaly, displaced and altered NPC proportions, increased number of IPCs, increased apoptosis	Johnson et al. (2018)
					Human cerebral organoid	9. RNAi knockdown of *ASPM* (co-electroporating GFP with shRNAs) and patient-derived cerebral organoids	9. Reduced organoid size, proliferation defect, reduced number of RGs and oRGs	Li et al. (2017)
MCPH6	Centromeric protein J	*CENPJ*	13q12.2	609279	Fly	1. Mutations in the *DSas-4* gene (P-element insertion)	1. Morphologically normal, no detectable centrioles or centrosomes, lack of cilia, early postnatal lethality	Basto et al. (2006)
						2. Point mutations	2. Centriole loss, reduced binding affinity of the DSas-4 and Ana2 interaction	Cottee et al. (2013)
					Rodent	3. Conditional *Cenpj* knockout in mouse brain	3. Long cilia and abnormal cilia disassembly, uncompleted cell division, reduced cell proliferation, increased apoptosis	Ding et al. (2019)
						4. *Cenpj*-knockout mice (cassette insertion between exons 4 and 5)	4. Microcephaly, dwarfism, skeletal abnormalities, increased levels of DNA damage, and apoptosis	McIntyre et al. (2012)
MCPH7	SCL/TAL1-interrupting locus protein	*STIL*	1p33	181590	Fish	1. Morpholino-mediated knockdown of *wdr62*	1. Reduction in head and eye size, prometaphase delay, increased apoptosis	Novorol et al. (2013)
						2. *Cassiopeia* (*csp*) mutant zebrafish	2. Embryonic lethality, high mitotic index, highly disorganized mitotic spindles, lack of centrosomes, increased apoptosis	Pfaff et al. (2007)
					Rodent	3. *Stil*-knockout mice (removal of exons 3–5)	3a. Embryonic lethality, defected neural folding, randomization of left-right asymmetry, impaired response to Sonic 3b. Hedgehog (SHH) signaling	Izraeli et al. (1999)
							Lack of centrioles and primary cilia	David et al. (2014)
MCPH8	Centrosomal protein 135 kD	*CEP135*	4q12	611423	Alga	1. *bld10* flagella-less mutants (insertional mutagenesis)	1. Lack of basal bodies, disorganized mitotic spindles and cytoplasmic microtubules, abnormal cell division, and slow growth	Matsuura et al. (2004)
						2. *bld10* null mutants (series of truncations)	2. Basal-body defects	Hiraki et al. (2007)
					Protozoa	3. SiRNA knockdown of *bld10* in *Paramecium*	3. Abnormal basal body assembly	Jerka-Dziadosz et al. (2010)
					Fly	4. *bld10*-knockout flies (transposon insertion)	4a. Disrupted localization of the inner and outer centriole components	Roque et al. (2012)
							4b. Short centrioles and basal bodies, immotile sperm, infertility	Mottier-Pavie and Megraw, (2009)
							4c. Lack of singlet microtubules and disassembly of central microtubule pair	Carvalho-Santos et al. (2012)
						5. *plp* RNAi knockdown in *bld10* mutant flies	5. Spindle alignment and centrosome segregation defects, perturbed centrosome asymmetry, mispositioned microtubule-organizing centers (MTOCs)	Singh et al. (2014)
MCPH9	Centrosomal protein 152 kD	*CEP152*	15q21.1	613529	Fly	1. *asterless* (*asl* ^*1*^, *asl* ^*2*^, *asl* ^*3*^) mutant flies (P-element-mediated transformation)	1. Defect in PCM stabilization and centrosome segregation, reduced microtubule nucleation, severe defects in meiotic spindle assembly	Varmark et al. (2007)
						2. *asterless* (*asl* ^*mecD*^) mutant flies (P-element-mediated transformation)	2. Lack of centrioles, basal bodies, and cilia	Blachon et al. (2008)
					Fish	3. Morpholino-mediated knockdown of *cep152*	3. Curly tail (ciliary defects)	
MCPH10	Zinc finger protein 335	*ZNF335*	20q13.12	610827	Rodent	1. *Znf335*-knockout mice (gene trap)	1. Early embryonic lethality	Yang et al. (2012)
						2. Conditional *Znf335* knockout in mouse brain (flanked promoter and exon1/2)	2. Lack all cortical structure and cortical neurons, enlarged ventricles	
						3. shRNA knockdown of *Znf335* in mice (*in utero* electroporation)	3. Disrupted NPC proliferation, premature differentiation, abnormal cell RGs orientation, disorganized dendritic outgrowth, lack of apical dendritic process	
MCPH11	Polyhomeotic-like 1 protein	*PHC1*	12p13.31	602978	N/A			
MCPH12	Cyclin-dependent kinase 6	*CDK6*	7q21.2	603368	Rodent	1. *Cdk6* knockout mice (removal of 1st coding exon)	1. Develop normally, slight hematopoiesis deficit	Malumbres et al. (2004)
MCPH13	Centromeric protein E	*CENPE*	4q24	117143	Fly	1. Mutations in *cenp-meta* gene (P-element-mediated disruption)	1. Embryonic lethality, defects in metaphase chromosome alignment	Yucel et al. (2000)
					Rodent	2. Conditional and complete *Cenp-e* gene disruptions in mouse	2. Early developmental arrest, mitotic chromosome misalignment	Putkey et al. (2002)
MCPH14	SAS-6 centriolar assembly protein	*SASS6*	1p21.2	609321	Worm	1. RNAi knockdown of *sas-6* in *Caenorhabditis elegans*	1. Abnormal centrosome duplication cycle	Leidel et al. (2005)
					Fish	2. *cellular atoll* (*cea*) mutant zebrafish	2. Defects in nuclear division, mitotic spindle formation, and centrosome duplication	Yabe et al. (2007)
					Fly	3. *sas-6*-knockout flies	3. Lack of cohesion between centrioles	Rodrigues-Martins et al. (2007)
MCPH15	Major facilitator superfamily domain-containing protein 2A	*MFSD2A*	1p34.2	614397	Fish	1. Morpholino-mediated knockdown of *mfsd2a*	1. Embryonic lethality before neural maturation, disrupted blood–brain barrier (BBB) integrity	Guemez-Gamboa et al. (2015)
					Rodent	2. *Mfsd2a*-knockout mice (gene targeting)	2a. Increased plasma lysophosphatidylcholine (LPC)	
							2b. Reduced body weight and length, increased energy expenditure, increased BAT β-oxidation, increased ataxic movement	Berger et al. (2012)
							2c. Reduced levels of DHA in the brain, microcephaly, neuronal cell loss in hippocampus and cerebellum, cognitive deficits, and severe anxiety	Nguyen et al. (2014)
							2d. Specific reduction in the retinal outer rod segment length, disorganized outer rod segment discs, reduction and mislocalization of rhodopsin, activated microglia	Wong et al. (2016)
						3. *Mfsd2a*-knockout mice (gene trap)	3. Leaky BBB, dramatic increase in central nervous system (CNS) endothelial cell vesicular transcytosis	Ben-Zvi et al. (2014)
						4. Mfsd2a-endothelial-specific knockout mice	4. Reduced neuronal arborization and decreased dendrite length	Chan et al. (2018)
MCPH16	Ankyrin repeat- and lem domain-containing protein 2	*ANKLE2*	12q24.33	616062	Worm	1. *ax475* mutant worms (missense mutation in the *lem-4L* open reading frame (ORF)) and RNAi knockdown of *lem-4L* in *C. elegans* embryos	1. Abnormal nuclear morphology	Asencio et al. (2012)
					Fly	2. *Ankle2* ^*A*^ knockout (ethyl methanesulfonate (EMS) chemical mutagenesis)	2. Loss of thoracic bristles, severe reduction in neuroblast, impaired cell proliferation, increased apoptosis	Yamamoto et al. (2014)
						3. *Ankle2* ^*A*^ knockout (EMS chemical mutagenesis) and Ankle2^CRIMIC^ knockout (CRISPR-Cas9)	3. Disrupted endoplasmic reticulum and nuclear envelope morphology, spindle alignment defects, disrupted asymmetric cell division pathway	Link et al. (2019)
MCPH17	Citron rho-interacting serine/threonine kinase	*CIT*	12q24.23	605629	Fly	1. *dck* ^*2*^ knockout (EMS chemical mutagenesis)	1. Defective in both neuroblast and spermatocyte cytokinesis, abnormal F actin and anillin rings	Naim et al. (2004)
					Rodent	2. *Flathead* (*fh*) mutant rats (spontaneous mutation, deletion within exon 1 of *Citron-K*)	2a. Reduced brain size, dysgenesis of neocortex, hippocampus, cerebellum, and retina, increased apoptosis, seizures, tremor, impaired coordination, and premature death	Roberts et al. (2000)
							2b. Reduced brain size, cytokinesis failure, binucleated cells	Sarkisian et al. (2002)
							2c. Decrease in the number of interneurons, hypertrophied soma and dendritic arbors of interneurons, increased apoptosis, cytokinesis failure, binucleated cells	Sarkisian et al. (2001)
						3. Citron-K-knockout mice (gene targeting)	3. Depletion of microneurons in the olfactory bulb, hippocampus, and cerebellum, increased apoptosis, abnormal cytokinesis, tremor and severe ataxia, reduced life span due to lethal epilepsy	Di Cunto et al. (2000)
MCPH18	WD repeat and FYVE domain-containing 3	*WDFY3*	4q21.23	617485	Fly	1. Blue cheese (*bchs*) knockout flies (P-element-mediated disruption)	1a. Extensive neurodegeneration, premature adult death, formation of protein aggregates, neuronal apoptosis	Finley et al. (2003)
							1b. Morphological abnormalities in motor neurons, increased apoptosis, reduced endolysosomal vesicles mobility	Lim and Kraut, (2009)
						2. *hALFY* mutant flies (single point mutation)	2. Reduced brain volume, very fragile and malformed brain, clusters of disorganized neurons, severe rough eye phenotype	Kadir et al. (2016)
					Rodent	3. *Disconnected* mutant mice (*Wdfy3* ^*disc/disc*^; spontaneous nonsense mutation in exon 59 of 67 of *Wdfy3*)	3. Perinatal lethality, enlarged frontal cortical aspects, tangential expansion but lateral thinning of the neocortical neuroepithelium, focal cortical dysplasia, abnormal ganglionic eminences, enlarged ventricles, reduction in the size of the olfactory bulbs	Orosco et al. (2014)
						4. *Wdfy3*-knockout mice (*Wdfy3* ^*lacZ/lacZ*^; gene targeting)	4. Perinatal lethality, more drastic thinning and lengthening of the neocortex, focal cortical dysplasias	
						5. *Wdfy3*-haploinsufficiency mice (*Wdfy3* ^*+/lacZ*^; gene targeting)	5a. Deficiencies in mitochondrial function, defective mitophagy, accumulation of defective mitochondria, compromised fatty acid β-oxidation	Napoli et al. (2018)
							5b. Decreased mitochondrial localization at synaptic terminals, decreased synaptic density, defective brain glycophagy, cerebellar hypoplasia	Napoli et al. (2021)
							5c. Macrocephaly, deficits in motor coordination and associative learning, downregulation of the Wnt signaling pathway	Le Duc et al. (2019)
MCPH19	Coatomer protein complex, subunit beta 2 (beta prime)	*COPB2*	3q23	606990	Rodent	1. *Copb2*-knockout mice *Copb2* ^*Zfn/Zfn*^; Zinc-Finger nuclease mediated deletion within exon 12)	1. Early embryonic lethality before organogenesis	DiStasio et al. (2017)
						*Copb2*-knockout mice (*Copb2* ^*null/null*^; CRISPR-Cas9)		
						2. Mice homozygous for the patient mutation (*Copb2* ^*R254C/R254C*^; CRISPR-Cas9)	2. Viable and do not have cortical malformations	
						3. Mice heterozygous for the patient mutation and a null allele (*Copb2* ^*R254C/Zfn*^; CRISPR-Cas9)	3. Perinatal lethality, reduced brain size, reduction in layer V cortical neurons, increased apoptosis	
MCPH20	Kinesin family member 14	*KIF14*	1q32.1	611279	Fish	1. *kif14* mutant zebrafish (sa24165 mutant line Kettleborough et al. (2013))	1. Microcephaly, eye defects, body curvature, cardiac edema, glomerular cysts, high mitotic index, ciliopathy-like phenotypes	Reilly et al. (2019)
					Fly	2. Mutations in the *Klp38B* gene (P-element insertion)	2. Semi-lethality, abnormal cell cycle progression, failure of cytokinesis, rough eyes, missing bristles, abnormal abdominal cuticles	Ohkura et al. (1997)
					Rodent	3. *Laggard* (*lag*) mutant mice (spontaneous mutation, disruption within exon 5 of *Kif14*) and *Kif14* knockout mice (gene targeting)	3. Small head, tremor, ataxic gait, severe hypomyelination in the CNS, disrupted cytoarchitecture in the neocortex, hippocampus, and cerebellar cortex, increased apoptosis during late neurogenesis	Fujikura et al. (2013)
MCPH21	Non-SMC condensin I complex, subunit D2	*NCAPD2*	12p13.31	615638	Rodent	1. *Ncaph2* condensin II mutant mice (*Ncaph2* ^*I15N/I15N*^; ENU chemical mutagenesis)	1. Isolated T-lymphocyte developmental defect, reduced brain size, increased anaphase DNA bridge formation in apical NPCs, impaired chromosome segregation	Gosling et al. (2007), Martin et al. (2016)
MCPH22	Non-SMC condensin II complex subunit D3	*NCAPD3*	11q25	609276				
MCPH23	Non-SMC condensin I complex subunit H	*NCAPH*	2q11.2	602332				
MCPH24	Nucleoporin 37	*NUP37*	12q23.2	609264	*Xenopus*	1. Morpholino-mediated knockdown of *nup107*, *nup85*, or *nup133*	1. Disrupted glomerulogenesis	Braun et al. (2018)
					Fish	2. *nup107* or *nup85* knockout in zebrafish (CRISPR-Cas9)	2. Developmental anomalies, early lethality	
MCPH25	Trafficking protein particle complex subunit 14	*TRAPPC14*	7q22.1	618350	Fish	1. *map11* knockout in zebrafish (CRISPR-Cas9)	1. Microcephaly, decreased neuronal proliferation	Perez et al. (2019)
						2. Morpholino-mediated knockdown of *c7orf43*	2. Curved bodies, small eyes, ciliogenesis defects	Cuenca et al. (2019)
MCPH26	Lamin B1	*LMNB1*	5q23.2	150340	Rodent	1. *Lmnb1*-knockout mice (*Lmnb1* ^*∆/∆*^; gene trap)	1a. Perinatal lethality, abnormal lung development and bone ossification, abnormal skeleton and skull shape	Vergnes et al. (2004)
							1b. Perinatal lethality, absence of the cortical layering with reduced number of neurons, absence of lamination in the hippocampus, absence of cerebellar foliation, impaired neuronal migration, reduced NPC proliferation, solitary nuclear bleb in cortical neurons	Coffinier et al. (2011)
						2. Forebrain-specific *Lmnb1*-knockout mice (*Emx1-Cre Lmnb1* ^*fl/fl*^)	2. Very small cortex, low neuronal density, lack of upper cortical layers, nuclear blebs in embryonic neurons, nuclear membrane ruptures, increased apoptosis, asymmetric distribution of Lmnb2	Coffinier et al. (2011), Chen et al. (2019)
						3. *Lmnb1/Lmnb2-*knockout mice (*Lmnb1* ^*−/−*^ *Lmnb2* ^*−/−*^; gene targeting)	3. Defects in lungs, diaphragms, and brains, thinner cerebral cortex, disorganized cortical layers, impaired neuronal migration, altered cleavage plane orientation, increased cell cycle exit	Kim et al. (2011b)
MCPH27	Lamin B2	*LMNB2*	19p13.3	150341	Rodent			
						4. *Lmnb2*-knockout mice (*Lmnb2* ^*−/−*^; gene targeting)	4. Perinatal lethality, impaired neuronal migration, layering defects in the cerebral cortex and hippocampus, absence of cerebellar foliation, absence of a discrete Purkinje cell layer, elongated nuclei in cortical neurons	Coffinier et al. (2010), Coffinier et al. (2011)
						5. Forebrain-specific *Lmnb2*-knockout mice (*Emx1-Cre Lmnb2* ^*fl/fl*^)	5. Small cortex, cortical defect more pronounced after birth, abnormal layering of cortical neurons, elongated nuclei in embryonic neurons, normal distribution of Lmnb1 at the nuclear rim	Coffinier et al. (2011)
MCPH28	Ribosomal RNA processing 7 homolog A	*RRP7A*	22q13.2	619449	Fish	1. *rrp7a* mutant zebrafish (sa11429 mutant line (Kettleborough et al., 2013))	1. Premature lethality, reduced brain size, reduced eye size, increased apoptosis	Farooq et al. (2020)

Classically, radiological investigations of patients with MCPH fail to show severe brain malformation except for simplified neocortical gyration. However, the increasing number of reported MCPH-linked mutations reveals that further deformities in brain architecture might occur (Table 2). The overall aim of this review is to explore the various effects of *MCPH* disease-causing genes on the cytoarchitecture of the cerebral cortex.

**TABLE 2:** Brain malformations associated with microcephaly primary hereditary (MCPH) additional to microcephaly.

Locus	Gene	Brain malformations	Key findings	Ref
MCPH1	*MCPH1*	Corpus callosum abnormalities	Mild hypoplasia	Hosseini et al. (2012)
			Agenesis of the genu	Neitzel et al. (2002)
		Pachygyria	+	Neitzel et al. (2002), Trimborn et al. (2004)
			Thickening of fronto-parietal and temporal gyri	Hosseini et al. (2012)
		Heterotopia	Nodular neuronal heterotopia (ventricular, infratentorial, and subependymal)	Neitzel et al. (2002), Trimborn et al. (2004)
			Periventricular neuronal heterotopias	Trimborn et al. (2004)
		Frontal lobe hypoplasia	+	Neitzel et al. (2002)
		Ventricular system abnormalities	Dilatation of lateral ventricles (dorsal and temporal), dilated external liquor space	
		Myelination/white matter abnormalities	Slight retardation of myelination of cerebral medullary layer	
MCPH2	*WDR62*	Corpus callosum abnormalities	Hypoplasia	Bilgüvar et al. (2010), Yu et al. (2010), Farag et al. (2013), Memon et al. (2013), Sgourdou et al. (2017), Zombor et al. (2019), Slezak et al. (2021)
			Dysplasia	Poulton et al. (2014)
			Abnormally shaped corpus callosum, agenesis of the rostral part	Bilgüvar et al. (2010)
			Dysmorphic with a thick body and a small genu	Yu et al. (2010)
			Incomplete genu and small splenium	
			Thinning of the corpus callosum with absence of the splenium	Banerjee et al. (2016)
		Pachygyria	+	Bilgüvar et al. (2010), Bhat et al. (2011), Bacino et al. (2012), Bastaki et al. (2016), Slezak et al. (2021)
			Diffuse pachygyria	Sgourdou et al. (2017), Zombor et al. (2019)
			Severe pachygyria	Poulton et al. (2014)
		Lissencephaly/agyria	Microlissencephaly	Bilgüvar et al. (2010), Bhat et al. (2011), Bastaki et al. (2016), Slezak et al. (2021)
		Polymicrogyria	+	Bilgüvar et al. (2010), Poulton et al. (2014)
			Widespread polymicrogyria	Yu et al. (2010)
			Polymicrogyria in the right hemisphere	Slezak et al. (2021)
			Bilateral polymicrogyria	Bhat et al. (2011)
			Bilateral parietal polymicrogyria	Murdock et al. (2011)
			Extensive polymicrogyria in the left cerebral hemisphere	
			Extensive areas of polymicrogyria in the right frontal lobe	Nardello et al. (2018)
		Abnormal Sylvian fissure	Under‐opercularization	Bilgüvar et al. (2010), Poulton et al. (2014)
			Widened Sylvian fissure	Farag et al. (2013)
			Open Sylvian fissures	Bacino et al. (2012)
		Schizencephaly	+	Memon et al. (2013)
			Open-lip schizencephaly	Bilgüvar et al. (2010)
			Narrow right temporoparietal open lip schizencephaly, right temporoparietal open lip schizencephaly	Yu et al. (2010)
			Suspected schizencephaly in the right parietal lobe	Banerjee et al. (2016)
			Closed schizencephaly in the right cerebral hemisphere	Slezak et al. (2021)
		Heterotopia	Subcortical heterotopia, bilateral band heterotopia in the posterior frontal and parietal lobes	Yu et al. (2010)
			Band heterotopias	Bhat et al. (2011)
			A focus of gray matter heterotopia in the right parietal region	Murdock et al. (2011)
		Hemispherical asymmetry	Asymmetric microcephalic hemispheres	Bilgüvar et al. (2010), Zombor et al. (2019)
			Volume loss worse on the left than the right cerebral hemisphere	Yu et al. (2010), Murdock et al. (2011)
			Volume loss worse on the right than the left cerebral hemisphere	Kousar et al. (2011), Rupp et al. (2014), Slezak et al. (2021)
		Frontal lobe hypoplasia	+	Farag et al. (2013)
		Hippocampal abnormalities	Dysmorphic	Bilgüvar et al. (2010)
			Simplified hippocampal gyration	Farag et al. (2013)
			Dysplasia of the temporal lobe with small hippocampus	Banerjee et al. (2016)
		Infratentorial abnormalities	Unilateral cerebellar hypoplasia, unilateral brainstem atrophy	Bilgüvar et al. (2010)
			Cerebellar hypoplasia	Farag et al. (2013)
			Slight atrophy of the brain stem and cerebellum	Banerjee et al. (2016)
		Ventricular system abnormalities	Dilated ventricles	Yu et al. (2010), Memon et al. (2013)
			Prominent extra-axial cerebrospinal	Kousar et al. (2011)
			Slight expansion of bilateral brain ventricles, obvious expansion of the fourth ventricle	Banerjee et al. (2016)
			Asymmetrical enlargement of the ventricles, dilated Virchow–Robin spaces	Zombor et al. (2019)
			Posterior horn-dominant enlargement of the lateral ventricles	Slezak et al. (2021)
		Thickened gray matter	+	Poulton et al. (2014), Zombor et al. (2019), Slezak et al. (2021)
			Diffusely thickened cortex	Bilgüvar et al. (2010)
			Mildly thickened cortex (∼5 mm)	Nicholas et al. (2010)
		Blurred gray-white matter junction	Loss of gray–white junction	Bilgüvar et al. (2010)
			Ill-defined gyral and nuclei pattern	Kousar et al. (2011)
			Indistinct gray–white matter border in certain areas	Zombor et al. (2019)
			Gray–white matter blurring involving the left parietooccipital cortex	Nardello et al. (2018)
		Myelination/white matter abnormalities	Leukodystrophy, dysplasia of the white matter	Banerjee et al. (2016)
			Thin white matter	Zombor et al. (2019)
			Reduced white matter volume	Slezak et al. (2021)
MCPH3	*CDK5RAP2*	Corpus callosum abnormalities	Hypoplasia	Alfares et al. (2018)
			Agenesis/hypogenesis	Issa et al. (2013)
		Hypothalamic abnormalities	Interhypothalamic adhesion	Nasser et al. (2020)
		Thickened gray matter	+	Alfares et al. (2018)
		Myelination/white matter abnormalities	Bilateral enhancement in the white matter (white matter disorder)	
MCPH4	*KNL1*	Infratentorial abnormalities	Cerebellar vermis hypoplasia	Saadi et al. (2016)
		Ventricular system abnormalities	Wide cyst in the posterior fossa communicating with an expanded fourth ventricle	
MCPH5	*ASPM*	Corpus callosum abnormalities	Thick corpus callosum	Passemard et al. (2009), Saadi et al. (2009), Létard et al. (2018)
			Agenesis of splenium	Passemard et al. (2009)
			Agenesis of rostrum	
			Partial agenesis	Abdel-Hamid et al. (2016)
			Hypoplasia	Abdel-Hamid et al. (2016), Létard et al. (2018)
		Pachygyria	+	Létard et al. (2018), Shaheen et al. (2019)
			Temporal pachygyria	Ariani et al. (2013)
		Polymicrogyria	Extensive unilateral perisylvian polymicrogyria from the frontal pole to the occipital pole	Passemard et al. (2009)
			Extensive bilateral posterior polymicrogyria	Létard et al. (2018)
			Polymicrogyria in frontoinsular region	
		Frontal lobe hypoplasia	Severe hypoplasia of the frontal lobes	Saadi et al. (2009)
			Frontal lobes are short and hypoplastic	Desir et al. (2008)
		Infratentorial abnormalities	Mild asymmetric cerebellar hypoplasia	Passemard et al. (2009)
			Ipsilateral pons hypoplasia	
			Cerebellar vermis and/or hemispheres hypoplasia	Abdel-Hamid et al. (2016), Létard et al. (2018)
			Elongated superior cerebellar peduncles	Létard et al. (2018)
			Relatively small pons	Abdel-Hamid et al. (2016)
			Thin brain stem	Ariani et al. (2013), Létard et al. (2018)
		Ventricular system abnormalities	Occipital horns of the lateral ventricles enlarged	Passemard et al. (2009)
			Dysmorphic frontal ventricles	Passemard et al. (2009)
			Enlarged lateral ventricles and colpocephaly	Abdel-Hamid et al. (2016)
			Large porencephalic cyst communicating with lateral ventricle	
			Small midline cyst	
			Ventricular enlargement	Létard et al. (2018)
			Arachnoid cyst in the posterior fossa	
			Enlarged Virchow–Robin spaces	
			Enlarged subarachnoid spaces, mega cisterna magna	
		Myelination/white matter abnormalities	Reduced white matter	Abdel-Hamid et al. (2016)
			Myelination delay	Létard et al. (2018)
MCPH6	*CENPJ*	N/A		
MCPH7	*STIL*	Corpus callosum abnormalities	Partial agenesis of the corpus callosum	Mouden et al. (2015), Shaheen et al. (2019)
			Short dysmorphic corpus callosum	Kakar et al. (2015)
		Holoprosencephaly	Lobar holoprosencephaly	Kakar et al. (2015), Mouden et al. (2015)
		Frontal lobe hypoplasia	Disproportionately short frontal lobes, continuity of the right and left frontal lobes at the level of the basal ganglia and lateral ventricles	Kakar et al. (2015)
			Straight and atrophic frontal lobe	Cheng et al. (2020)
		Infratentorial abnormalities	Atrophy of the vermis	Mouden et al. (2015)
			Cerebellar hypovermis dysplasia	Cheng et al. (2020)
		Ventricular system abnormalities	Absence of ventricular frontal horns	Mouden et al. (2015)
			Absence of occipital lobe and a large unilateral temporal and occipital fluid cavity communicating	
			Small third ventricle, enlarged lateral ventricles posteriorly	Kakar et al. (2015)
			Large porencephalic cyst replacing most of the posterior right hemisphere	
			Dilatation of the fourth ventricle	Cheng et al. (2020)
		Blurred gray-white matter junction	+	Cheng et al. (2020)
		Myelination/white matter abnormalities	Diffuse severe reduction of the white matter volume	Kakar et al. (2015)
MCPH8	*CEP135*	Heterotopia	Bilateral nodular heterotopia in the peritrigonal regions	Bamborschke et al. (2020)
MCPH9	*CEP152*	Corpus callosum abnormalities	Severe hypogenesis	Shaheen et al. (2019)
		Polymicrogyria	+	
		Ventricular system abnormalities	Inter-hemispheric cyst at left aspect of the falx continuous with the third ventricle	
MCPH10	*ZNF335*	Corpus callosum abnormalities	Thin corpus callosum	Stouffs et al. (2018)
		Lissencephaly/agyria	Anterior agyria and a posterior simplified gyral pattern	
		Basal ganglia abnormalities	Absent basal ganglia	Stouffs et al. (2018)
			Invisible basal ganglia	Sato et al. (2016)
			Volume loss in basal ganglia (putamen atrophy)	Caglayan et al. (2021)
			Patchy areas of the altered signal in the left thalamoganglionic region	Rana et al. (2019)
		Infratentorial abnormalities	Hypoplasia of brainstem and cerebellum	Sato et al. (2016), Stouffs et al. (2018)
			Cerebellar atrophy	Rana et al., (2019), Caglayan et al. (2021)
		Ventricular system abnormalities	Enlarged ventricles	Stouffs et al. (2018)
		Myelination/white matter abnormalities	Hypomyelination	Sato et al. (2016), Stouffs et al. (2018), Caglayan et al. (2021)
MCPH11	*PHC1*	N/A		
MCPH12	*CDK6*			
MCPH13	*CENPE*	Corpus callosum abnormalities	Partial agenesis of the corpus callosum	Mirzaa et al. (2014)
		Frontal lobe hypoplasia	Low forehead	
		Infratentorial abnormalities	Cerebellar hypoplasia	
		Lissencephaly/agyria	Diffuse severely simplified gyral pattern with virtually no gyri over the frontal lobe	
		Myelination/white matter abnormalities	Immature white matter	
MCPH14	*SASS6*	Lissencephaly/agyria	No gyral or sulcal development	Zhang et al. (2019b)
		Basal ganglia abnormalities	Poorly confined basal ganglia and missing delineation of the internal capsule	Khan et al. (2014)
		Infratentorial abnormalities	Dysmorphic infratentorial region with hypoplasia of the vermis cerebella	
		Ventricular system abnormalities	No bilateral frontal horns or cavum septi pellucidi present	Zhang et al. (2019b)
			Abnormal formation of the lateral ventricles	Khan et al. (2014)
MCPH15	*MFSD2A*	Corpus callosum abnormalities	Hypoplasia	Guemez-Gamboa et al. (2015), Scala et al. (2020)
		Infratentorial abnormalities	Inferior vermian hypoplasia	Scala et al. (2020)
			Pontine hypoplasia	
			Hypoplasia of brain stem and cerebellum	Guemez-Gamboa et al. (2015)
		Ventricular system abnormalities	Enlarged ventricles	Guemez-Gamboa et al. (2015), Harel et al. (2018), Scala et al. (2020)
			Hydrocephaly	Shaheen et al. (2019)
		Myelination/white matter abnormalities	White matter reduction	Alakbarzade et al. (2015), Harel et al. (2018), Scala et al. (2020)
MCPH16	*ANKLE2*	Corpus callosum abnormalities	Agenesis	Yamamoto et al. (2014)
			Partial agenesis	Shaheen et al. (2019)
		Pachygyria	Coarsening of the gyral sulcal pattern and some thickening consistent with pachygyria	
		Polymicrogyria	Polymicrogyria-like cortical brain malformations	Yamamoto et al. (2014)
		Infratentorial abnormalities	Hypoplastic cerebellum	Shaheen et al. (2019)
		Ventricular system abnormalities	Small frontal horns of the lateral ventricles with mildly enlarged posterior horns	Yamamoto et al. (2014)
		Thickened gray matter	Mildly thickened cortex	
MCPH17	*CIT*	Corpus callosum abnormalities	Hypogenesis	Li et al. (2016a)
			Agenesis	Harding et al. (2016), Shaheen et al. (2016)
		Lissencephaly/agyria	Lissencephaly	Harding et al. (2016), Shaheen et al. (2016)
		Infratentorial abnormalities	Cerebellar and brainstem hypoplasia	Harding et al. (2016)
		Ventricular system abnormalities	Enlarged ventricles	Harding et al. (2016), Shaheen et al. (2016)
		Myelination/white matter abnormalities	Diminished white matter	Shaheen et al. (2016)
MCPH18	*WDFY3*	N/A		
MCPH19	*COPB2*	Corpus callosum abnormalities	Thin corpus callosum	DiStasio et al. (2017)
		Ventricular system abnormalities	Slight dilation of the lateral, third and fourth ventricles	
		Myelination/white matter abnormalities	Delayed myelination	
MCPH20	*KIF14*	Corpus callosum abnormalities	Agenesis	Moawia et al. (2017), Reilly et al. (2019)
			Partial agenesis	Makrythanasis et al. (2018)
		Lissencephaly/agyria	Microlissencephaly	Makrythanasis et al. (2018)
			+	Moawia et al. (2017)
		Infratentorial abnormalities	Cerebellar hypoplasia	Moawia et al. (2017), Reilly et al. (2019)
		Ventricular system abnormalities	Large basal cisterns	Makrythanasis et al. (2018)
			Interhemispheric cyst	Moawia et al. (2017)
		Thickened gray matter	Slightly thickened cortex	Moawia et al. (2017)
MCPH21	*NCAPD2*	N/A		
MCPH22	*NCAPD3*			
MCPH23	*NCAPH*			
MCPH24	*NUP37*	Infratentorial abnormalities	Cerebellar vermis hypoplasia	Braun et al. (2018)
MCPH25	*TRAPPC14*	Corpus callosum abnormalities	Hypoplasia	Perez et al. (2019)
		Myelination/white matter abnormalities	Diminished white matter	
MCPH26	*LMNB1*	Corpus callosum abnormalities	Thin corpus callosum	Cristofoli et al. (2020)
			Dysgenesis	
		Pachygyria	+	
		Lissencephaly/agyria	Lissencephaly	
		Ventricular system abnormalities	Enlarged ventricles	
MCPH27	*LMNB2*	Ventricular system abnormalities	Enlarged ventricles	Parry et al. (2021)
		Myelination/white matter abnormalities	Diminished white matter	
MCPH28	*RRP7A*	Corpus callosum abnormalities	Volume loss especially in the anterior half	Farooq et al. (2020)

## Normal Corticogenesis

MCPH arises principally from a decreased production of neurons due to defects in progenitor proliferation, differentiation, and/or apoptosis during critical stages of brain development. Hence, it is important to briefly review the normal process of cortical neurogenesis before discussing the multiple facets of MCPH protein functions in maintaining a smooth running of this process.

Before the neurogenesis journey begins, the neural stem cells represented by neuroepithelial progenitors (NE) at the ventricular zone (VZ) undergo initial expansion in number through symmetrical cell divisions (Homem et al., 2015). Once the antiproliferative gene Tis21 starts to be expressed, NE cells begin to switch from proliferative division to neuronic division (Götz and Huttner, 2005). Simultaneously, NE cells transform gradually into more fate-restricted progenitors known as radial glial cells (RGCs) as an indication for their glial gene expressions (Götz and Huttner, 2005; Mori et al., 2005; Subramanian et al., 2019). RGCs possess apical processes attaching to the ventricular surface and basal processes reaching the basement membrane (future pial surface) (Homem et al., 2015). RGCs expand their number and exhibit a much higher number of asymmetrical cell divisions as compared with NE cells (Subramanian et al., 2019). During cell expansion, RGC nuclei show a characteristic interkinetic nuclear migration (INM) synchronized with the cell cycle phases during proliferation (Kosodo et al., 2011). The RGC nuclei migrate toward the basal side of the developing cortex during G1 phase and remain there during S phase before they migrate apically during G2 phase and proceed with M-phase once they reach the ventricular surface (Kosodo et al., 2011; Miyata et al., 2014). This pattern of migration during early neurogenesis requires functional microtubules and actin filaments (Götz and Huttner, 2005). It has been proposed that INM allows RGC rapid proliferation while maintaining their dense packing and determines cell fate through signaling gradients along their migration pathway (Götz and Huttner, 2005; Baye and Link, 2007; Del Bene et al., 2008). It is therefore very likely to find defects in neurogenesis involving RGC expansion and neuronal cell fate decisions when INM is disrupted (Latasa et al., 2009). Intriguingly, INM shows differences between species and might affect the total number of the generated neurons and thence the brain size (Okamoto et al., 2014).

Asymmetrical division of an RGC at the ventricular surface generates a self-renewing RG daughter cell and either a postmitotic neuron or a basal progenitor (intermediate progenitor cell (IPC)) (Homem et al., 2015; Subramanian et al., 2019). It has been earlier believed that the asymmetrical division of RGCs is principally driven by a change in mitotic spindle positions leading to a shift of the cleavage plane orientation from perpendicular (vertical) to parallel (horizontal) relative to the ventricular surface (Chenn and McConnell, 1995; Zhong et al., 1996; Kosodo et al., 2004; Fietz and Huttner, 2011; Homem et al., 2015). However, further investigations revealed that the rate of asymmetrical divisions in RGCs is not necessarily altered by the orientation of the cleavage plane (Morin et al., 2007; Konno et al., 2008; Postiglione et al., 2011). The asymmetrical RGC fate might be affected by inheriting centrioles with different maturity and primary cilium (Wang et al., 2009; Goetz and Anderson, 2010; Paridaen et al., 2013). It has been also shown that alterations in RGC cycle length control the shift from self-renewing divisions to neurogenic divisions (Calegari and Huttner, 2003; Calegari et al., 2005; Pilaz et al., 2009; Arai et al., 2011). Furthermore, it has been proposed that Notch signaling triggers neurogenic cell fate either by its distinct apicobasal gradient during INM or through asymmetric inheritance of endosomes positive for Sara (Smad anchor for receptor activation) (Del Bene et al., 2008; Nerli et al., 2020). This latter is achieved by targeting signaling endosomes to the central spindle by the action of plus-end kinesin motor (Klp98A) (Derivery et al., 2015).

Unlike RGCs, IPCs lack the connection with the ventricular surface and settle mainly in the subventricular zone (SVZ) basal to the VZ (Rakic, 2009; Homem et al., 2015; Subramanian et al., 2019; Heide and Huttner, 2021). IPCs undergo some proliferative divisions and terminate by generating two cortical neurons (Noctor et al., 2004; Pontious et al., 2008; Rakic, 2009). Asymmetrical divisions of RGCs in many mammals, especially in primates, yield an additional generation of a special type of basal progenitors known as basal RGCs (outer RGCs (oRGCs)) (Homem et al., 2015). Compared with IPCs, the oRGCs show a much higher proliferative capacity, which amplifies the total number of generated neurons and contribute to the characteristic folded cerebral cortex observed in primates, especially in humans (Reillo et al., 2011; Fietz et al., 2010; Hansen et al., 2010; Betizeau et al., 2013; Heide and Huttner, 2021). The newly established 3D *in vitro* human brain organoid model exhibits a considerable number of oRGCs (Lancaster et al., 2013; Zhang et al., 2019a).

Postmitotic cortical neurons are generated from both VZ and SVZ neural progenitors in an inside-out manner by which later-born neurons (superficial layers IV–II) bypass earlier-born neurons (deep layers VI–V) (Molyneaux et al., 2007; Leone et al., 2008). This pattern of neural generation is under spatiotemporal, cell cycle, and competency precise controls of neural progenitor fates (Kohwi and Doe, 2013). Eventually, six neural layers are created in the developing cerebral cortex, and the neurons start the formation of their distinctive dendrites, axons, and functional synapses. The fully developed cortical network contains 80% glutamatergic excitatory neurons produced by VZ and SVZ neural progenitors located in the dorsal telencephalon and 20% GABAergic inhibitory neurons that originated from the medial and caudal ganglionic eminence (Marín, 2013; Costa and Müller, 2014). The terminal number of generated cortical neurons is affected not only by the original number of neural progenitors but also by their starting and ending proliferation points and their cell lineage (Homem et al., 2015).

At the final stage of neurogenesis, RGCs lose their neuronal lineage and the connection with the apical surface switching to glial cell generators (Malatesta et al., 2000; Qian et al., 2000). Cortical astrocytes are firstly detected followed by oligodendrocytes, and the number of both glial cells is hugely expanded postnatally (Qian et al., 2000). Glial cells induce the development of white matter and axonal outgrowth by producing myelin and forming astrocytic branches (Subramanian et al., 2019). Taken together, forming a normal cerebral cortex requires highly organized spatiotemporal control for the neural progenitor populations to generate different neuronal and glial subtypes. Any defect during this process can lead to a major impact on brain development.

## Brain Phenotype in Individuals With Microcephaly Primary Hereditary

The morphological changes in the brain structure of MCPH individuals have been mainly identified by radiological studies. Most MCPH cases show a reduction in brain volume associated with a simplified neocortical gyration pattern. However, the increased number of reported mutations and the ongoing neuroimaging of MCPH individuals reveal further brain malformations (Table 2). Some of these structural changes point toward the causative *MCPH* gene (e.g., the association between malformations of basal ganglia and mutations in gene encoding zinc finger-335 protein (*ZNF335*; *MCPH10*) (Sato et al., 2016; Stouffs et al., 2018; Rana et al., 2019; Caglayan et al., 2021)). The increasing number of reported brain malformations in MCPH individuals widens its pathogenesis spectrum. This indicates that the disruption of MCPH proteins not only is affecting the generation of neurons but could additionally affect neuronal differentiation, migration, dendritic and axonal outgrowth, and synaptogenesis. This is understandable given the fact that MCPH proteins are highly expressed in various neuroprogenitor organelles, especially the centrosome. In the following sections, we will discuss the consequences of *MCPH* mutations on brain development.

## Accidents During the Brain Development Journey in Microcephaly Primary Hereditary

**FIGURE 1:**
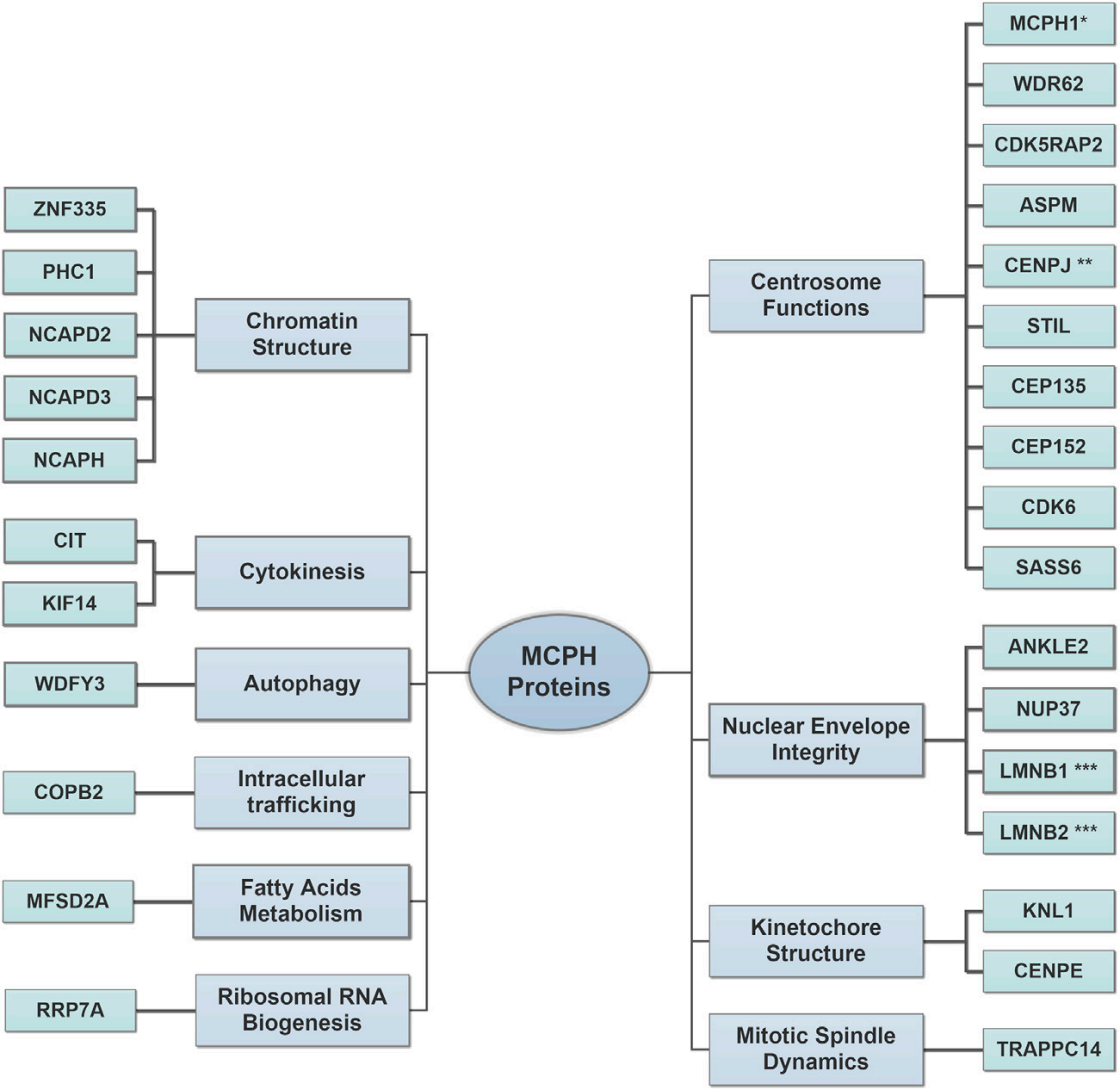
Major roles of microcephaly primary hereditary (MCPH) proteins in brain development. The increased number of discovered MCPH proteins expands the pathomechanism spectrum to include several cellular components. Centrosome Functions: the proteins of this group regulate proper centrosomal functions to balance the transition between neural progenitor cell (NPC) proliferation and differentiation by controlling cell cycle progression and cell cycle exit fraction. Nuclear Envelope Integrity: the proteins of this group affect the proper spindle alignment and cell fate determinants during NPC proliferation and protect radial glial cell (RGC) nuclei from mechanical stress injury during INM. Kinetochore Structure: the proteins of this group assure the correct alignment of chromosomes during mitosis. Mitotic Spindle Dynamics: the proteins of this group regulate the spindle dynamics and cell division. Chromatin Structure: the proteins of this group regulate gene expression during neurogenesis and assure proper DNA damage repair. Cytokinesis: the proteins of this group regulate the terminal step in the cell cycle, which leads to a physical separation between the daughter cells. Autophagy: the proteins of this group facilitate the removal of cytosolic protein aggregates and maintain mitochondrial homeostasis. Intracellular trafficking: the proteins of this group control the cellular retrograde trafficking from the Golgi to the endoplasmic reticulum. Fatty Acid Metabolisms: the proteins of this group affect the postnatal neuronal morphogenesis, which requires a normal lipogenesis process. Ribosomal RNA Biogenesis: the proteins of this group regulate ribosomal RNA processing and affect primary cilia resorption. Please refer to (Table 1) for full protein names. *MCPH1 is also involved in chromatin structure. **CENPJ is also involved in kinetochore structure. ***LMNB1 and LMNB2 are also involved in mitotic spindle dynamics.

Studying the molecular mechanisms behind the pathogenesis of MCPH is very limited in humans. In fact, *MCPH* genes are highly conserved among different species (Woods et al., 2005; Gilbert et al., 2005), and this led to the discovery of several MCPH animal models mimicking the human phenotype (Table 1). Therefore, most of our current knowledge on the role of MCPH proteins in brain development is enriched through extensive studies on MCPH animal models. However, the pronounced difference of the human brain compared with most of the studied MCPH animal models establishes a new research direction toward 3D *in vitro* human brain organoid systems in studying the pathogenesis of microcephaly (Muzio and Consalez, 2013; Gabriel et al., 2020). The remarkable presence of oRGCs in this model opens the door for deeper insights into their role during the course of this disease in humans (Lancaster et al., 2013; Zhang et al., 2019a). As many MCPH proteins share overlapped functions, we saw to categorize them according to their major role(s) rather than discussing each one individually (Figure 1).

### Dysfunctional Centrosome

Almost one-third of *MCPH* mutations occur in centrosomal or mitotic spindle proteins. Defective centrosomes can affect cell cycle progression and cell division, leading to abnormal chromosomal numbers, cell cycle arrest, and apoptosis (Barbelanne and Tsang, 2014). It has been proposed that alterations of the cleavage plane orientation during NE proliferation increase asymmetric cell divisions (Buchman and Tsai, 2007; Knoblich, 2008; Zhong and Chia, 2008). This, in turn, leads to an early consumption of progenitor cells at the expense of a premature generation of neurons with ultimately reduced number, thence a smaller brain (Fish et al., 2006; Buchman et al., 2010; Kaindl et al., 2010; Lizarraga et al., 2010). In this notion, several MCPH mouse models show a shift in the cleavage plan orientation of NE cells favoring neurogenic cell fate (Fish et al., 2006; Lizarraga et al., 2010; Pulvers et al., 2010; Gruber et al., 2011; Xu et al., 2014). This evidence has been also supported by human brain organoid models (Lancaster et al., 2013; Zhang et al., 2019a). Intriguingly, most of the MCPH fly models with defected centrosomal proteins exhibit normal brain size (Basto et al., 2006; Lucas and Raff, 2007; Roque et al., 2012), indicating that changes in the cleavage plane orientation might only have a minor impact on brain growth. Alternatively, flies could have compensatory mechanisms bypassing the effect of the misoriented cleavage plane (Nigg and Raff, 2009). On the other hand, further studies discovered MCPH mouse models with the microcephaly phenotype, though unaffected cleavage plane (Pulvers et al., 2010; Marjanović et al., 2015). Furthermore, depletion of some other MCPH centrosomal proteins in mice does not affect brain growth at all (Malumbres et al., 2004).

The neural progenitor cell (NPC) symmetrical proliferation speed is frequently reduced in mutated *MCPH* genes, which encode centrosomal proteins (Lizarraga et al., 2010; Gruber et al., 2011; Sgourdou et al., 2017; Ding et al., 2019). This is much obvious toward the end of neurogenesis (Lizarraga et al., 2010; Gruber et al., 2011; Sgourdou et al., 2017) when the later-born neurons (superficial layers II–IV) start to be generated. Together with the premature generation of neurons, this explains why superficial cortical neurons are the most affected in most MCPH models (Lizarraga et al., 2010; Chen et al., 2014). This is in line with postmortem histological analysis described in a case of *WD-repeat-containing protein 62 gene* (*WDR62*; *MCPH2*) mutation (Yu et al., 2010). In addition, MCPH proteins are important for the normal distribution of cells between cortical zones. Knockout of *abnormal spindle-like*, *microcephaly-associated gene* (*Aspm*) in ferret increases the number of generated oRGCs, affecting the RGC overall proliferative capacity (Johnson et al., 2018). Likewise, knockdown of the cyclin-dependent kinase five regulatory subunit-associated protein two gene *Cdk5rap2* in a mouse model alters the distribution of progenitor pool leading to more generation of basal progenitors (Buchman et al., 2010). By contrast, somatosensory cortical layer VI has been reported to be thinner in an *Aspm* knockout mouse model (Fujimori et al., 2014). Indeed, several *in vivo* and *in vitro* studies including human brain organoid models revealed that *MCPH* centrosomal genes balance the transition between NPC proliferation and differentiation by controlling cell cycle progression and cell cycle exit fraction (Buchman et al., 2010; Lizarraga et al., 2010; Bogoyevitch et al., 2012; Lancaster et al., 2013; Hainline et al., 2014; Zhang et al., 2019a). This explains, respectively, the reduced proliferation and premature neuronal differentiation detected in the respective MCPH animal models. Furthermore, using the conditional knockout mouse model, it has been shown that centromeric protein J (Cenpj) regulates NPC cell cycle progression by regulating cilium disassembly during neurogenesis (Ding et al., 2019). Similarly, depletion of *WDR62* and centrosomal-P4.1-associated protein (*CPAP*) in human cerebral organoids impairs the cilium disassembly and cell cycle progression (Gabriel et al., 2016; Zhang et al., 2019a).

It has been reported that mutations in genes encoding MCPH centrosomal proteins alter the maturation and cellular number of centrosomes (Pfaff et al., 2007; Rodrigues-Martins et al., 2007; Yabe et al., 2007; Megraw et al., 2011; Hussain et al., 2012; McIntyre et al., 2012; Hussain et al., 2013; Arquint and Nigg, 2014). This, in turn, might affect the proper distribution of chromosomes to daughter cells leading to spindle instability and mitotic delay or arrest at metaphase checkpoint (Ripoll et al., 1985; Gonzalez et al., 1990; Megraw et al., 1999; Riparbelli et al., 2002; Matsuura et al., 2004; Pfaff et al., 2007; Lizarraga et al., 2010; Kim et al., 2011a; Vitale et al., 2011; Novorol et al., 2013; Chen et al., 2014). In most of these cases, such defect triggers the apoptotic cascade leading to cellular loss (Pfaff et al., 2007; Lizarraga et al., 2010; Vitale et al., 2011; McIntyre et al., 2012; Novorol et al., 2013; Chen et al., 2014). Remarkably, increased apoptosis of NPCs—associated with or without proliferation/differentiation defects—contributes to the microcephaly phenotype by depleting the neural stem cell pool (Izraeli et al., 1999; Pfaff et al., 2007; Lizarraga et al., 2010; Gruber et al., 2011; McIntyre et al., 2012; Sgourdou et al., 2017; Zhang et al., 2019a; Ding et al., 2019). Intriguingly, neuronal populations also seem to be vulnerable to apoptosis during later stages of development (Lizarraga et al., 2010).

Apparently, the impact of mutated MCPH centrosomal genes in brain development not only is restricted to NPC proliferation and differentiation phase but also exceeds it to affect neuronal migration, dendritic and axonal outgrowth, and synaptogenesis. The presence of gray matter heterotopia, polymicrogyria, lissencephaly, and pachygyria in several MCPH conditions points toward impaired neuronal migration (Table 2) (Leventer et al., 2010; Yu et al., 2010). The signs of this impairment have been reported in postmortem histopathological MCPH samples and various MCPH animal models (Yu et al., 2010; Buchman et al., 2011; Xu et al., 2014). Besides that, an interesting role of Cdk5rap2 in regulating dendritic development and synaptogenesis in superficial cortical layer II/III has been reported (Zaqout et al., 2019). The contribution of dendritic complexity deficits in the microcephaly phenotype points toward a progressive nature during the MCPH course (van Dyck and Morrow, 2017).

### Defective Chromatin Structure

Chromatin structure is a golden stone for gene expression regulation during neurogenesis. Mutations in genes encoding chromatin-linked proteins expand the pathomechanism spectrum of the MCPH. *Microcephalin* (*BRCT/BRIT1*) mutated cells taken from MCPH1 individuals and mouse models displayed premature chromosome condensation (PCC) associated with a high frequency of prophase-like cells and defective DNA damage repair (Jackson et al., 2002; Neitzel et al., 2002; Liang et al., 2010; Trimborn et al., 2010). This feature has been considered as a diagnostic marker for individuals with MCPH1 gene mutations (Jackson et al., 2002). In flies, *mcph1* mutants display embryonic lethality due to mitotic arrest and uncoordinated centrosome/nuclear cycles in early syncytial cell cycles (Brunk et al., 2007). Likewise, mutations in gene encoding *polyhomeotic-like one protein* (*PHC1*; *MCPH11*) are associated with aberrant DNA damage repair (Awad et al., 2013). In addition, *PHC1* mutations disturb the expression of Nanog and the ubiquitination of histone H2A, in which the former maintains pluripotency and the latter affects the cell cycle progression by the accumulation of Geminin (Awad et al., 2013; Chen et al., 2021).

Complete ablation of *Znf335* gene in mice is embryonically lethal, but conditional knockout leads to a reduction in the cortical size affecting the forebrain much severely (Yang et al., 2012). This has been attributed to disruptions in NPC proliferation, cell fate, and neuronal differentiation (Yang et al., 2012). Consistently, postmortem histopathological studies on brain samples taken from ZNF335 patients reveal a severe reduction in the neuronal number associated with abnormalities in neuronal morphogenesis, migration, and polarity (Yang et al., 2012).

Mutations in genes encoding condensin complex proteins NCAPD2, NCAPD3, and NCAPH have been linked to MCPH21, 22, and 23, respectively (Martin et al., 2016). One of the hallmarks of hypomorphic Ncaph2 mice is the formation of a chromatin bridge in apical NPCs (Martin et al., 2016). These bridges result from failed sister chromatid disentanglement leading to chromosome segregation errors and aneuploidy (Martin et al., 2016). Subsequently, NPCs undergo a reduced cell proliferation and an increased apoptosis without obvious alterations in spindle orientations or cell fat (Martin et al., 2016).

### Deformed Kinetochore Proteins

Mutations in genes encoding kinetochore scaffold one protein (KNL1, previously known as CASC5) and centromere-associated protein E (CENPE) have been linked to MCPH4 (Jamieson et al., 1999; Genin et al., 2012; Mirzaa et al., 2014; Saadi et al., 2016; Szczepanski et al., 2016; Zarate et al., 2016). Proper function of proteins associated with centromeric kinetochore assures the correct alignment of chromosomes during mitosis; else, spindle assembly checkpoint (SAC) is activated and suspends the mitotic progression (Cleveland et al., 2003; Musacchio and Salmon, 2007; Santaguida and Musacchio, 2009; Hori and Fukagawa, 2012). Conditional knockout of *Knl1* in mouse cortical NPCs results in DNA damage due to chromosomal segregation errors (Shi et al., 2019). This triggers a p53-dependent apoptotic cascade and leads to massive loss of NPCs and microcephaly (Shi et al., 2019). Similar to MCPH centrosomal proteins, the progressive loss of NPCs in *Knl1* conditional knockout mice affects mainly the later-born neurons (superficial layers II–IV) (Shi et al., 2019). CENPE facilitates the transition from metaphase to anaphase during the cell cycle (Yen et al., 1991). Disruption of Cenpe function in mice and *Drosophila* leads to early embryonic lethality due to chromosomal instability (Yucel et al., 2000; Putkey et al., 2002).

### Interruption of Fatty Acids Uptake Into the Brain

Proper brain development and function require essential omega-3 fatty acids, which need to be obtained from the circulation via specific transporters (Alakbarzade et al., 2015). Sodium-dependent lysophosphatidylcholine (LPC) transporter (MFSD2A) is exclusively expressed in the endothelium of the blood–brain barrier (BBB) and a major transport facilitator for docosahexaenoic acid (DHA) (Ben-Zvi et al., 2014; Nguyen et al., 2014; Alakbarzade et al., 2015; Guemez-Gamboa et al., 2015). Depending on the transporter residual activity, mutations in *MFSD2A* (*MCPH15*) gene is associated with either a progressive microcephaly syndrome or a much lethal phenotype (Berger et al., 2012; Alakbarzade et al., 2015; Guemez-Gamboa et al., 2015; Harel et al., 2018; Scala et al., 2020). The progressive feature associated with the milder form of this disease raises the possibility that LPC transportation is continuously required for membrane biogenesis in the brain (Alakbarzade et al., 2015; Scala et al., 2020). Endothelial-specific deletion of *Mfsd2a* in mice leads to a microcephaly phenotype accompanied by a reduction in neuronal arborization and dendritic length (Chan et al., 2018). Interestingly, neuronal loss detected in *Mfsd2a* knockout mice was restricted to cerebellar Purkinje cells and hippocampal CA1 and CA3 regions (Nguyen et al., 2014). Taken together, these data demonstrate that, unlike other MCPHs, Mfsd2a deficiency affects the postnatal neuronal morphogenesis, which requires a normal lipogenesis process (van Deijk et al., 2017; Ziegler et al., 2017; Chan et al., 2018). Notably, variable degrees of white matter reduction have been also reported in *MCPH15* individuals (Alakbarzade et al., 2015; Harel et al., 2018; Scala et al., 2020). Further studies are required to assess to which extent do white matter deficits contribute to the microcephaly phenotype (Huang and Li, 2021).

### Altered Nuclear Envelope

Mutations in several genes encoding nuclear envelop components have been recently linked to MCPH conditions. Ankyrin repeat- and lem domain-containing protein 2 (Ankle2) is localized to the endoplasmic reticulum and nuclear envelope (Link et al., 2019). *Drosophila dAnkle2* mutant larvae show a reduction in the brain size due to impaired nuclear envelope integrity, which eventually affects proper spindle alignment and cell fate determinants during NPC proliferation (Link et al., 2019). Another study, however, suggests that the reduction in *Drosophila dAnkle2* mutant NPCs cells is due to defects in proliferation and massive apoptosis rather than an alteration in asymmetrical cell division (Yamamoto et al., 2014). In this line, *Caenorhabditis elegans* ANKLE2 ortholog protein (LEM-4L) plays a critical role in mitosis by facilitating nuclear envelope reassembly during mitotic exit (Asencio et al., 2012).

Individuals with mutations in genes encoding various nuclear pore complex proteins (NPC) are diagnosed with severe forms of nephrotic syndrome (Braun et al., 2018). However, mutations in NPC subunit component nucleoporin 37 (NUP37) also exhibit intellectual disability and MCPH (Braun et al., 2018). More recently and yet to be linked to a specific OMIM MCPH number, mutations in NUP85 subunit are associated with a reduction in brain volume, delayed myelination, agenesis of the corpus callosum, gray matter heterotopia, and frontal lobe cortical malformation (Ravindran et al., 2021). Fibroblasts derived from NUP85 individuals are characterized by reduced cell viability, proliferation rate, abnormal mitotic spindle apparatus, and altered cytoskeletal protein expressions (Ravindran et al., 2021). As most of the studies performed in viable animal models with NPC defects focused on the nephrotic phenotype, further investigations to understand their effects on brain growth are still warranted.

B-type lamins 1 and 2 (LMNB1/2) are intermediate filament proteins involved in nuclear envelope reassembly, in which the deficiency leads to fragile nuclei more susceptible to nuclear membrane (NM) rupture (Coffinier et al., 2011; Chen et al., 2019). In humans, mutations in LMNB1 and LMNB2 have been linked to MCPH26 and MCPH27, respectively (Cristofoli et al., 2020; Parry et al., 2021). During early neurogenesis, RGC nuclei undergo INM, which represents mechanical stress that threatens RGCs with weakened nuclear lamina (Chen et al., 2019). Therefore, lack of murine Lmnb1/2 during this critical step triggers NPC apoptosis and leads to abnormal neuronal migration reflected by disorganized cortical layering (Kim et al., 2011b; Coffinier et al., 2011; Young et al., 2012; Chen et al., 2019). This migration defect not only is confined to the cerebral cortex but also affects the hippocampal and cerebellar layering (Coffinier et al., 2010; Coffinier et al., 2011). In addition, it has been proposed that Lmnb1/2 is localized at the mitotic spindle and plays a role in INM and neuronal migration via interaction with dynein in organizing NPC spindle orientation (Tsai et al., 2006; Kim et al., 2011b). However, no abnormal metaphase spindle formation has been noticed in lymphoblastoid cells (LCLs) derived from LMNB1 individuals (Cristofoli et al., 2020).

### Defective Cytokinesis

Cytokinesis is the terminal step in the cell cycle, which leads to a physical separation between the daughter cells. Defects in this process frequently result in the formation of binucleated cells, aneuploidy, chromosomal instability, cell cycle arrest, and apoptosis (Li et al., 2016a). Notably, the elevated number of binucleated cells—including pyramidal and Purkinje cells—is considered as a key feature for cytokinesis failures (Di Cunto et al., 2000; Roberts et al., 2000; Reilly et al., 2019). Citron rho-interacting kinase (CIT) midbody protein has important roles in cytokinesis, and its defect leads to MCPH17 in humans (Li et al., 2016a; Basit et al., 2016; Harding et al., 2016; Shaheen et al., 2016). Postmortem histopathological analysis of brain samples taken from *MCPH17* individuals reveals a thickened neocortex with disorganized layers and unmyelinated white matter with scattered neurons (Harding et al., 2016). In addition, the cerebellar cortex and hippocampus show dysplastic and hypoplastic features, and Purkinje cells exhibit a simplified dendritic tree where many of them are multinucleated (Harding et al., 2016). Studies conducted in *Cit* knockout rodent models reveal that NPCs undergo massive apoptosis due to interrupted cytokinesis (Di Cunto et al., 2000; Roberts et al., 2000). In these models, binucleated neurons have been detected in several brain and spinal cord regions; however, apoptosis seems to be more pronounced in the cerebral cortex, granular layers of cerebellum, hippocampus, and olfactory bulb (Di Cunto et al., 2000; Sarkisian et al., 2002). In addition, the high rate of cell death reported at the ganglionic eminence reduces the total number of generated interneurons (Sarkisian et al., 2001; Di Cunto et al., 2000). Consistent with human brain findings, cerebellar Purkinje cells are disorganized and show underdeveloped dendritic complexity (Di Cunto et al., 2000). The presence of disorganized cortical layering and scattered neurons in the white matter should raise the possibility of an abnormal migration process even though it is yet to be confirmed by further studies.

The role of CIT in cytokinesis requires a proper function of kinesin family 14 (KIF14) microtubule motor protein (Makrythanasis et al., 2018). It is then unsurprising to realize that mutations in *KIF14* also lead to MCPH by a common mechanism as *CIT* mutations (Moawia et al., 2017; Makrythanasis et al., 2018; Reilly et al., 2019). This is supported by several studies conducted in various animal models with depleted *KIF14* homologs (Ohkura et al., 1997; Fujikura et al., 2013; Reilly et al., 2019). Mutations of *Drosophila KIF14* homolog, also known as kinesin-like protein at 38B (*KLP38B*), affect the cell cycle progression due to cytokinesis failure (Ohkura et al., 1997). In the same notion, *Laggard* mice (*lag*), an animal model for KIF14, are characterized by microcephaly, cortical dysgenesis, and severe hypomyelination as a consequence of massive apoptosis during late neurogenesis (Fujikura et al., 2013). Consequently, Cux1-positive upper cortical neurons are much reduced in number, and some of them are displaced (Fujikura et al., 2013). Similar to *Cit* knockout models, *lag* mice show scattered cerebellar Purkinje cells with simplified dendritic trees pointing toward abnormalities in neuronal migration and neurite formation (Fujikura et al., 2013).

### Disturbed Autophagy and Mitochondrial Dynamics

MCPH18 is caused by mutations in gene encoding *WD repeat and FYVE domain-containing 3* (*WDFY3*), also known as *Autophagy-Linked FYVE* (*ALFY*) (Kadir et al., 2016). Normally, this scaffolding protein facilitates the removal of cytosolic protein aggregates, which, in turn, maintains mitochondrial homeostasis (Kadir et al., 2016; Napoli et al., 2018; Napoli et al., 2021). Wdfy3 is highly expressed in RGCs, and its loss of function prevents the transition from symmetrical proliferative divisions to asymmetrical differentiative divisions by altering the Wnt signaling cascade (Tacchelly-Benites et al., 2013; Orosco et al., 2014; Kadir et al., 2016). The imbalance in NPCs mode of cell division leads to regional differences in neocortical thickness and opposing phenotypes of micro- and macrocephaly (Orosco et al., 2014; Le Duc et al., 2019). On the other hand, the disruption of mitochondrial dynamics in *Wdfy3* mutant mice decreases the synaptic density, alters the synaptic plasticity, and probably affects dendritic development (Napoli et al., 2018; Napoli et al., 2021). Remarkably, proteins involved in GABAergic neurotransmission are downregulated in *Wdfy3* mutant mice (Napoli et al., 2021). Furthermore, Wdfy3 plays a role in neural migration during early neurogenesis (Orosco et al., 2014).

### Interrupted Intracellular Trafficking

Coatomer Protein Complex Subunit Beta 2 (COPB2) controls the cellular retrograde trafficking from the Golgi to the endoplasmic reticulum (Orci et al., 1993; Letourneur et al., 1994). Interestingly, mutations in *COPB2* interrupt brain growth and lead to MCPH19 (DiStasio et al., 2017). While the complete loss of Copb2 is incompatible with life, partial loss of Copb2 in mice interferes with the growth of the brain (DiStasio et al., 2017). This has been associated with increased cell death and a high number of proliferative cells positive for phosphorylated histone H3 (pH3) (DiStasio et al., 2017). Still, further studies are necessitated to dissect the exact role of Copb2 in controlling brain size.

### Disturbed Mitotic Spindle Dynamics

Trafficking protein particle complex subunit 14 (TRAPPC14)—also known as microtubule-associated protein 11 (MAP11)—is localized to mitotic spindles and interacts with α-tubulin regulates the spindle dynamics and cell division (Perez et al., 2019). Recently, *TRAPPC14* mutations have been linked to MCPH25 in human and microcephaly phenotypes in the zebrafish model (Perez et al., 2019). This has been mainly attributed to a decreased brain cell proliferation due to altered spindle dynamics affecting the mitotic progression and probably the cytokinesis, however, without increased apoptosis (Perez et al., 2019). In addition, TRAPPC14 has been implicated in ciliogenesis and cilia stability, which, in turn, could affect brain growth (Cuenca et al., 2019).

### Defective Ribosome Biogenesis

The most recently diagnosed MCPH28 cases have been linked to a mutation in *Ribosomal RNA Processing seven Homolog A* (*RRP7A*) (Farooq et al., 2020). It is known that mutations in genes involved in ribosome biogenesis are associated with neurodevelopmental defects together with other abnormalities (Hetman and Slomnicki, 2019). The encoded RRP7A protein shows high expression in RGCs and cellular localizations at the centrosome, primary cilium, and nucleolus (Farooq et al., 2020). Depletion of RRP7A alters ribosomal RNA processing and affects primary cilia resorption, causing a delay in S-phase entry and progression (Farooq et al., 2020). Mutated rrp7a zebrafish embryos display a reduction in the expression pattern of some proliferation and neural differentiation markers, while TUNEL assay analysis indicates increased apoptosis (Farooq et al., 2020). These findings might result from defective rrp7a functions at the level of centrosome and/or primary cilia. MicroRNA processing has been identified as a contributing factor in temporal fate specification (Kohwi and Doe, 2013). Thus, dysregulated ribosomal RNA processing with subsequent nucleolar stress establishes a new insight into MCPH pathomechanisms.

## Microcephaly Primary Hereditary Versus Infection-Induced Microcephaly

After describing the genetic component of microcephaly, we here shed some light on some infectious agents associated with microcephaly. Particularly *Toxoplasma gondii*, cytomegalovirus, rubella virus, and syphilis, but also the herpes simplex virus, HIV, and Zika virus, have been reported in children born with microcephaly. The severity of microcephaly depends not only on the type of the infectious agent but much importantly on the gestational age when an infection occurs (Devakumar et al., 2018). It has been shown that neural progenitors are targeted by these pathogens; however, the mechanisms by which most of these infections lead to microcephaly are not fully understood (Devakumar et al., 2018). Nevertheless, the epidemic infections with the Zika virus and its association with congenital microcephaly triggered extensive research in this field. Several studies based on various *in vitro* and *in vivo* models point toward NPC cell cycle arrest or an increase in cell death upon the infection with the Zika virus (Adams Waldorf et al., 2016; Li et al., 2016b; Cugola et al., 2016; Garcez et al., 2016; Tang et al., 2016; Wu et al., 2016). Intriguingly, several MCPH genes including *Mcph1*, *Aspm*, *Cdk5rap2*, *Stil*, and *Cep135* are downregulated in brain tissues extracted from Zika-infected mice (Li et al., 2016b; Wu et al., 2016). This raises the possibility that infection-induced microcephaly might alter brain growth via altering the expression of various MCPH genes. However, the direct impact of infectious agents on the pathogenesis of microcephaly cannot be ruled out.

## Conclusion

**FIGURE 2:**
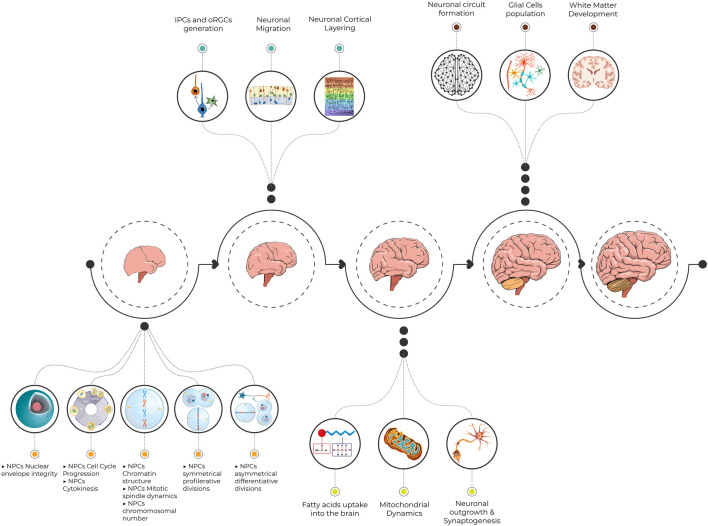
An illustrative figure demonstrating the pathway toward normal brain development. Minor defects at crucial steps in neurogenesis result in various neurodevelopmental disorders including MCPH. NPCs, neural progenitor cells; IPCs, intermediate progenitor cells; oRGCs, outer radial glial cells; MCPH, microcephaly primary hereditary.

The journey during brain growth and development is impeded at specific points with crucial steps (Figure 2). Minor defects at any of these developmental points result in various neurodevelopmental disorders including MCPH. The earlier the insult during the journey, the greater the impact on brain growth. The major obstacle is faced during the rapid NPC proliferation just before the commencing generation of neurons. Either decreased proliferation or increased NPCs apoptosis depletes the neuronal stem cell pool and ultimately leads to a smaller number of generated neurons. Obviously, most MCPHs are caused by mutations in centrosomal proteins. Hence, dysfunctional centrosome alters NPC proliferation, cell cycle progression, and cell fate determination. In addition, the resulting aneuploidy and increased DNA damage response associated with some mutated MCPH genes trigger apoptosis. The accelerated number of discovered MCPH genes expands the pathomechanism spectrum of this disease beyond the centrosomal component. Similarly, the simultaneous generation of animal models mimicking the human MCPH phenotype provides a strong platform for future studies to dissect further molecular mechanisms behind the microcephaly phenotype. This will also expand our knowledge of normal brain growth and evolution.
